# Immune Reconstitution Inflammatory Syndrome: Opening Pandora's Box

**DOI:** 10.1155/2017/5409254

**Published:** 2017-01-09

**Authors:** Mariana Meireles, Conceição Souto Moura, Margarida França

**Affiliations:** ^1^Internal Medicine Department, Porto Hospital Centre, Porto, Portugal; ^2^Pathological Anatomy Department, São João Hospital Centre, Porto, Portugal; ^3^Clinical Immunology Unit, Porto Hospital Centre, Porto, Portugal

## Abstract

One of the purposes of antiretroviral therapy (ART) is to restore the immune system. However, it can sometimes lead to an aberrant inflammatory response and paradoxical clinical worsening known as the immune reconstitution inflammatory syndrome (IRIS). We describe a 23-year-old male, HIV1 infected with a rapid progression phenotype, who started ART with TCD4+ of 53 cells/mm^3^ (3,3%) and HIV RNA = 890000 copies/mL (6 log). Four weeks later he was admitted to the intensive care unit with severe sepsis. The diagnostic pathway identified progressive multifocal leukoencephalopathy, digestive Kaposi sarcoma, and* P. aeruginosa* bacteraemia. Five weeks after starting ART, TCD4+ cell count was 259 cells/mm^3^ (15%) and HIV RNA = 3500 copies/mL (4 log). He developed respiratory failure and progressed to septic shock and death. Those complications might justify the outcome but its autopsy opened* Pandora*'s box: cerebral and cardiac toxoplasmosis was identified, as well as hemophagocytic syndrome, systemic candidiasis, and* Mycobacterium avium complex* infection. IRIS remains a concern and eventually a barrier to ART. Male gender, young age, low TCD4 cell count, and high viral load are risk factors. The high prevalence of subclinical opportunistic diseases highlights the need for new strategies to reduce IRIS incidence.

## 1. Background

Antiretroviral therapy (ART) led to a dramatic change in the clinical picture and prognosis of the Human Immunodeficiency Virus (HIV) infection. However, some patients develop a paradoxical worsening of their clinical status after starting therapy. HIV-associated immune reconstitution inflammatory syndrome (IRIS) has emerged as an important early complication of ART introduction, particularly in patients with severe immunosuppression. The diagnosis is based on an unexpected clinical worsening, days to months after the ART introduction, an abrupt rise of TCD4+ cell count, and a decrease >1 log in HIV RNA load in the presence of pathological antigens [1]. Mortality rate is around 5.4% [2] reaching up to 45% if concomitant opportunistic diseases occur. Early diagnosis and therapy are crucial to a favorable outcome but diagnosis of the leading opportunistic antigen can be challenging.

## 2. Case Presentation

A 23-year-old male was diagnosed with HIV infection in July 2011, having a negative HIV serology 6 months earlier. By September 2011, his TCD4+ cell count was 563 cells/mm^3^ (15%) with a HIV RNA of 88500 copies/mL. HBV, HCV, syphilis,* Mycobacterium tuberculosis*, CMV, and* Toxoplasma* screenings were negative and chest X-ray, abdominal ultrasound, and colonoscopy were unremarkable. During the follow-up, although presenting with a stable TCD4+ cell count, he kept high viral load and a serodiscordant sexual partner, those being reasons for initiating ART, which he refused. In February 2012 secondary syphilis was diagnosed with a TCD4+ count of 264 cells/mm^3^ (7,5%) and a HIV RNA load of 339000 copies/mL (5,5 log). Three months later, with 53 TCD4+ cells/mm^3^ (3.3%) and a viral load of 890000 copies/mm^3^ (6 log) he was started on TDF + FTC + EFV.

**Figure 1:**
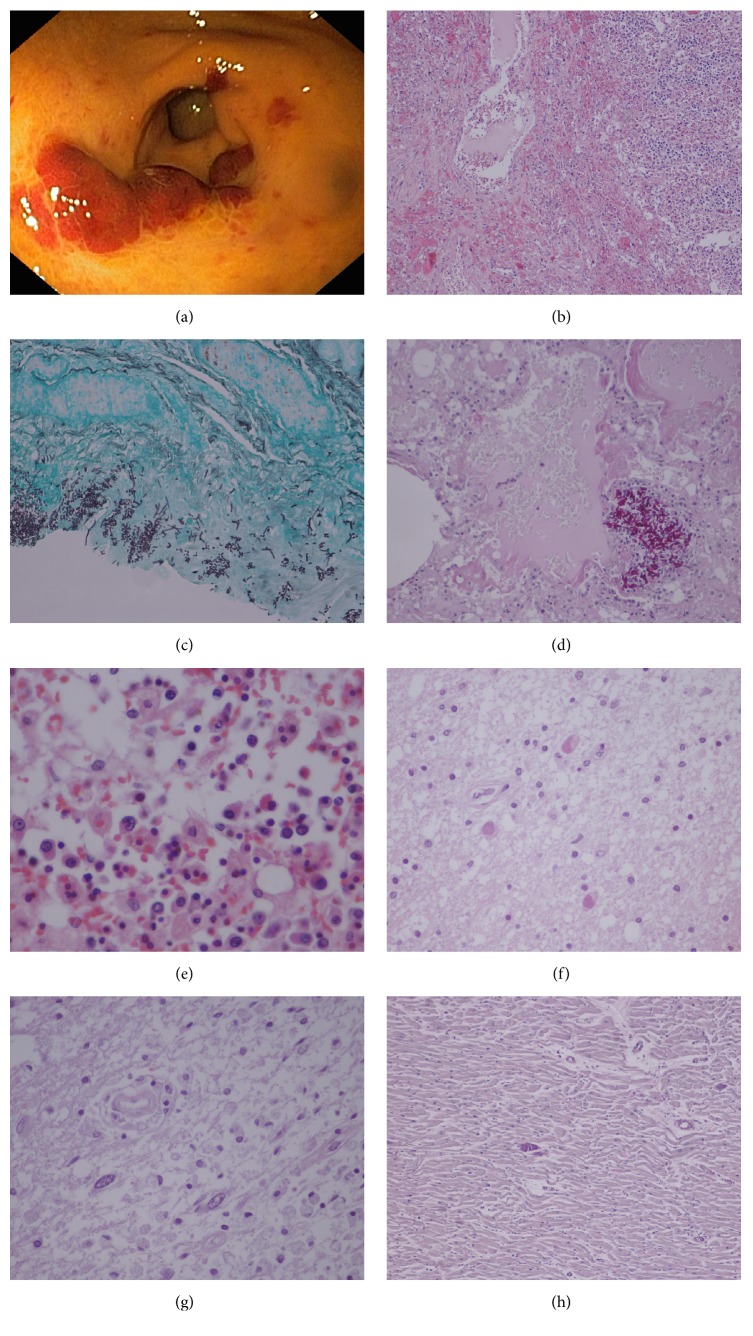
(a) Gastric Kaposi sarcoma: scarlet slightly elevated lesions of the antral gastric mucosal surface (upper gastrointestinal endoscopy). (b) Mediastinal ganglia Kaposi sarcoma: submucosal vascular spindle-shaped cells (H&E-stain, 100x). (c)* Candida* spp. in colon (Grocott methenamine silver-stain, 100x). (d)* Candida* spp. in lung and hyaline membranes suggesting acute respiratory distress syndrome (H&E-stain, 100x). (e) Bone marrow hemophagocytosis (H&E-stain, 400x). (f) Progressive multifocal leukoencephalopathy: enlarged homogeneous oligodendrocyte nucleus with inclusion (H&E-stain, 100x). (g) Cerebral toxoplasmosis:* Toxoplasma *cyst (H&E-stain, 100x). (h) Cardiac toxoplasmosis:* Toxoplasma *cyst (H&E-stain, 100x).

Four weeks later he was admitted to the emergency department with fever, oral candidiasis, diarrhea, hypotension, and pancytopenia. Gastrointestinal sepsis was suspected and he was started on ciprofloxacin and fluconazole. He developed shock and respiratory failure in the next 48 h and was admitted to the intensive care unit. Antibiotic regimen was changed to imipenem, metronidazole, and fluconazole. Faeces microbiological and parasitological tests were negative, blood and urine cultures were sterile, and CMV plasma antigen was negative. Five weeks after starting ART there was an increase in TCD4+ cell count [259 cells/mm^3^ (15%)] and a 2 log drop in the HIV viral load [3500 copies/mm^3^ (4 log)]. Initial thoracic, abdominal, and pelvic CT scan were unremarkable. Bronchial aspirate and bronchoalveolar lavage (BAL) were sterile for fungi and fast growing bacteria. PCR assays to identify* Chlamydophila*,* Legionella*,* Mycoplasma*, CMV, HSV, and* Mycobacterium tuberculosis* were negative. The patient remained febrile, and due to severe pancytopenia, hepatosplenomegaly, and an elevated ferritin, hemophagocytic syndrome was suspected, not being confirmed on the bone marrow aspirate though. By the 9th hospitalization day he presented with seizures and the MRI scan showed bilateral and multifocal white matter with high signal intensity on T2-weighted and FLAIR images. This, associated with the presence of JC virus in CSF, led to the diagnosis of progressive multifocal leukoencephalopathy (PML). Diarrhea persisted but due to clinical instability and severe anemia and thrombocytopenia, endoscopic studies were only performed on the 24th hospitalization day. Upper and lower endoscopy revealed multiple polypoid lesions with a cherry-red appearance in the stomach and colon, compatible with Kaposi sarcoma (Figure 1(a)). He was started on doxorubicin. Three days later, along with* Pseudomonas aeruginosa* bacteraemia, the clinical state deteriorated and the patient died.

The clinical course and the documented complications, some of them defining AIDS, would be sufficient to explain the poor outcome but the autopsy opened an unexpected underworld: Kaposi sarcoma was confirmed in the stomach and colon but also in the esophagus and mediastinal lymph nodes (Figure 1(b));* Candida* species was found in the anal canal, colon (Figure 1(c)), and lung, where hyaline membranes compatible with an acute respiratory distress syndrome were also seen (Figure 1(d)). As previously suspected, prominent phagocytosis of blood cells in the bone marrow confirmed hemophagocytic syndrome (Figure 1(e)); brain histology showed enlarged oligodendroglial cells nucleus with ground glass inclusions consistent with PML (Figure 1(f)); multiple basophilic dot-like parasites in cysts were documented in cerebral (Figure 1(g)) and heart tissues (Figure 1(h)) configuring cerebral and myocardial toxoplasmosis.* Mycobacterium avium complex* culture from the bronchial aspirate became positive after death.* A. baumannii *grew from the right atrium blood culture and aspects of anal condyloma and intraepithelial low-grade neoplasm were also identified, as well as several aspects consistent with systemic shock.

## 3. Discussion/Conclusion

Chronic HIV infection is a disease of coinfections with immunosuppression allowing reactivation of dormant pathogens or increasing susceptibility to exogenous ones. The vast majority of HIV-infected patients have one or more coinfections at some point during the disease course, which play an important role in chronic immune activation. The higher the antigen burden, the higher the risk for IRIS. This case presents multiple unmasking-IRIS with systemic Kaposi sarcoma, systemic candidiasis, and PML, coupled with several more or less quiescent defining AIDS diseases.

A meta-analysis with 13103 HIV-infected patients showed that IRIS occurred in 16.1% of the cases after starting ART [2]. This number can significantly increase in the presence of coinfections, reaching 45%. Male gender, young age, low TCD4+ cell count, and high viral load are all IRIS risk factors. It has been showed that low TCD4+ cell count and high plasma HIV RNA levels at the time of diagnosis are associated with a faster progression to AIDS [3]. At least 1 value of CD4+ <100 cells/mm^3^ in the first year of seroconversion seems to identify a rare group of individuals at high risk for faster disease progression [4]. The patient in this report had a decrease of almost 80% in TCD4+ cell count in 3 months. This subset of patients has the ability to collect several opportunistic infections (OI) in few months, before the risk for specific OI be identified and effective prophylaxis started.

Several factors are independently associated with occurrence of OI such as African American/Black race or Hispanic/Latino ethnicity, intravenous drug users, heterosexual HIV transmission, lower TCD4+ cell count, and higher viral load [5]. Sixteen large cohorts of HIV-infected patients were recently evaluated and reported a decrease in the incidence rates of first OIs, from 2.96 events/100 person-years in 2000–2003 to 1.45 events/100 person-years in 2008–2010 [5]. The improvements in viral suppression and immune status associated with newer ART regimens played a major role in the picture of OI. Levels of TCD4+ cell count below which specific OIs tend to occur were never absolute. Patients that started ART during 2008–2010 had a 7% probability of developing a new OI within 2 years when the initial TCD4+ cell count was <200 cell/mm^3^ but only 1% when the initial TCD4+ cell count was ≥500 cells/mm^3^ [5]. The median TCD4+ cell count is usually above or close to 200 cells/mm^3^ for tuberculosis and isosporiasis infections and less than 100 cells/mm^3^ for candidiasis, PCP, CMV infection, and MAC infection [5]. On the other hand, OIs also have an impact on TCD4+ cell count as the latter decrease at a rate of 24.1 cells/mL per three months in the presence of OI, compared to an increase of 21.3 cells/mL per three months in its absence [6]. In this report, the patient had a rapid decline in TCD4+ cells to 53 cells/mm^3^ leading to a profound immunosuppressed state, which dramatically increased the risk for several OI in a short period of time.

Along with depression of immune system, OI presentation becomes blurred and the diagnosis is difficult. Our patient, despite his profound immunosuppression, was asymptomatic until the start of ART. KS is the most common HIV-associated malignancy and disease exacerbation or presentation after starting ART can be as high as 29% [7]. Central nervous system IRIS contributes to the bulk of IRIS mortality: JC virus infection is the third cause after* Mycobacterium *tuberculosis and* Cryptococcus neoformans*; cerebral toxoplasmosis is rare. Twelve to 22% of AIDS patients had endomyocardial involvement by* T. gondii* at autopsy; in the highly active ART era the prevalence of cardiac toxoplasmosis confirmed* postmortem* has been reported to be less than 10% [8]. The incidence of* M. avium complex* infection related to IRIS, in the presence of TCD4+ <100 cells/mm^3^, is 3.5% [9].

Early HIV infection detection and treatment are crucial to a better prognosis. Recent changes in the treatment guidelines are expected to reduce IRIS incidence, particularly by reducing the duration and degree of immunosuppression. Nevertheless, systematic screening for OI before starting ART is still a key element to prevent this phenomenon. The risk of paradoxical IRIS could also be substantially altered by deferral of ART initiation, particularly in central nervous system IRIS. This approach is postulated to ensure adequate treatment of OI, reduce the antigen burden before starting ART in HIV-infected patients with severe immune deficiency, and promote immune recovery.

Despite the lack of standardized treatment protocol for IRIS, ART should not be interrupted and there may be a need for corticosteroid therapy. As such, treatment of these patients is a huge challenge and further research regarding the immunopathogenesis, diagnosis, and its management should be pursued.
